# One-year supplementation with *Lactobacillus reuteri* ATCC PTA 6475 counteracts a degradation of gut microbiota in older women with low bone mineral density

**DOI:** 10.1038/s41522-022-00348-2

**Published:** 2022-10-19

**Authors:** Peishun Li, Boyang Ji, Hao Luo, Daniel Sundh, Mattias Lorentzon, Jens Nielsen

**Affiliations:** 1 Department of Biology and Biological Engineering, Chalmers University of Technology, Gothenburg, Sweden; 2 BioInnovation Institute, Ole Maaløes Vej 3, DK2200 Copenhagen, Denmark; 3 Sahlgrenska Osteoporosis Centre, Institute of Medicine, Sahlgrenska Academy, University of Gothenburg, Gothenburg, Sweden; 4 Region Västra Götaland, Geriatric Medicine Clinic, Sahlgrenska University Hospital, Gothenburg, Sweden; 5 Mary MacKillop Institute for Health Research, Australian Catholic University, Melbourne, VIC 3000 Australia; 6 Novo Nordisk Foundation Center for Biosustainability, Technical University of Denmark, DK2800 Kgs. Lyngby, Denmark

**Keywords:** Microbiome, Microbiota

## Abstract

Recent studies have shown that probiotic supplementation has beneficial effects on bone metabolism. In a randomized controlled trial (RCT) we demonstrated that supplementation of *Lactobacillus reuteri* ATCC PTA 6475 reduced bone loss in older women with low bone mineral density. To investigate the mechanisms underlying the effect of *L. reuteri* ATCC PTA 6475 on bone metabolism, 20 women with the highest changes (good responders) and the lowest changes (poor responders) in tibia total volumetric BMD after one-year supplementation were selected from our previous RCT. In the current study we characterized the gut microbiome composition and function as well as serum metabolome in good responders and poor responders to the probiotic treatment as a secondary analysis. Although there were no significant differences in the microbial composition at high taxonomic levels, gene richness of the gut microbiota was significantly higher (*P* < 0.01 by the Wilcoxon rank-sum test) and inflammatory state was improved (*P* < 0.05 by the Wilcoxon signed-rank test) in the good responders at the end of the 12-month daily supplementation. Moreover, detrimental changes including the enrichment of *E. coli* (adjusted *P* < 0.05 by DESeq2) and its biofilm formation (*P* < 0.05 by GSA) observed in the poor responders were alleviated in the good responders by the treatment. Our results indicate that *L. reuteri* ATCC PTA 6475 supplementation has the potential to prevent a deterioration of the gut microbiota and inflammatory status in elderly women with low bone mineral density, which might have beneficial effects on bone metabolism.

## Introduction

Osteoporosis is a highly prevalent bone disease in the elderly population, and is characterized by decreased bone mineral density (BMD), deteriorated bone microarchitecture, reduced bone strength and increased susceptibility to fragility fractures. The risk of fracture can be reduced by pharmacological treatment, but treatment rates in patients with osteoporosis remain low, possibly due to low osteoporosis awareness, costs for medication and fear or rare side effects of presently available drugs^1,2^. Thus, there is an urgent need to develop a novel and effective intervention for the prevention and treatment of osteoporosis.

Towards this goal, the gut microbiota has been revealed to play pivotal roles in bone metabolism, potentially by regulating the immune system and osteoclast formation in mice^3–6^. This suggests that modulating the gut microbiome by nutritional supplements with probiotics may provide novel strategies for the prevention and treatment of osteoporosis. In earlier experimental studies, the supplementation of probiotic strain *Lactobacillus reuteri* ATCC PTA 6475 has been demonstrated to reduce bone loss and increase bone density in mice with estrogen deficiency or increased inflammation^7,8^. In our previous randomized controlled trial, we observed that supplementation of *L. reuteri* ATCC PTA 6475 could reduce bone loss by ~50% in older women with low BMD^9^. Thus, *L. reuteri* ATCC PTA 6475 may be a potential therapeutic strategy to prevent postmenopausal bone loss. However, we also observed that part of the subjects responded poorly to the treatment with *L. reuteri* ATCC PTA 6475 (i.e., poor responders with less relative changes in tibia total volumetric BMD)^9^.

Initial reports suggest that the effects of probiotic supplementations on bone metabolism might involve the modulation of the composition and function of the gut microbiota^10–12^. Specifically, different probiotics were reported to expand intestinal butyrate-producing bacteria^10^, indicating that probiotics may indirectly increase production of short chain fatty acids (SCFAs) by the gut microbiota. Moreover, the probiotic *Lactobacillus rhamnosus* GG has been suggested to increase bone formation by stimulating the production of microbial metabolite butyrate, which induced T cell-produced Wnt10b in eugonadic young mice^11^.

To gain mechanistic insight into the effect of *L. reuteri* ATCC PTA 6475 on bone metabolism and identify factors important for a good response to the probiotic, here we characterized the metagenomic profile of the fecal microbiota and the serum metabolome of good responders and poor responders, who had the highest and lowest changes in tibia total volumetric BMD in our randomized controlled trial^9^, respectively. After a one-year probiotic supplementation, we found decreased inflammation and significantly increased gene richness of the gut microbiota in the good responders, while altered microbial composition and function, including enrichment of *E. coli* and its biofilm formation in the poor responders. Our study reveals new possible mechanisms contributing to the regulation of bone loss in older women, which could be crucial for the development of novel osteoporosis treatments.

## Results

### Probiotic supplementation decreases inflammation and increases bone mineral density in the good responders

**Fig. 1:**
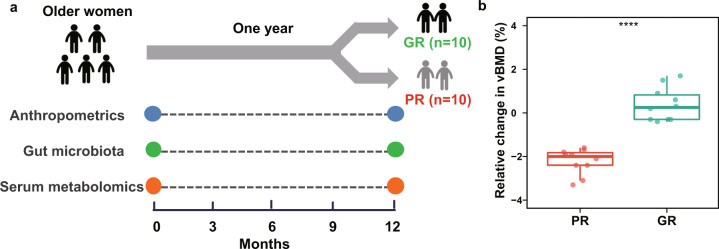
The scheme diagram of experimental design for elderly women with differential responses to the supplementation with *L. reuteri* ATCC PTA 6475. **a** Women with a good response (GR group, *n* = 10) and with a poor response (PR group, *n* = 10) were recruited. Serum samples and fecal samples were collected from the older women with bone loss at baseline and 12 months. The complete metabolomic profiling for these women were performed. However, for metagenomic profiling, one woman in the GR and PR group lost one fecal sample, respectively. Therefore, nine women had time points-paired fecal samples in each group. **b** The boxplot shows the relative change of tibia total volumetric BMD in the GR and PR groups after one-year treatment. ‘****’ denotes *P* < 0.001. The upper quartile, median and lower quartile are shown in the box plot.

To investigate differences in subjects with different responses to supplementation with the probiotic strain *L. reuteri* ATCC PTA 6475, we selected 20 elderly women by identifying ten women with a good response (GR group) and ten women with a poor response (PR group) from the per protocol (PP) population as previously described^9^ (Fig. 1a). The demographic and clinical characteristics of these 20 subjects at baseline and 12 months were shown in Table 1. There were no significant differences in these characteristics between the GR and PR groups at both time points, but subjects in the GR group (27.5 ± 3.6 kg/m^2^ (baseline) and 27.7 ± 3.7 kg/m^2^ (12 months)) had slightly higher body mass index (BMI) than the PR group (24.0 ± 2.8 kg/m^2^ (baseline) and 24.1 ± 3.1 kg/m^2^ (12 months)) (*P* < 0.05 by the *t*-test). After one-year treatment with *L. reuteri* ATCC PTA 6475, the relative change in tibia total volumetric BMD exhibited significantly increased levels in the GR group (0.39 ± 0.77) compared to the PR group (−2.22 ± 0.58; *P* < 0.001 by the *t*-test; Fig. 1b). In addition, relative changes in cortical thickness and cortical volumetric BMD differed between the two groups (*P* < 0.001 using a *t*-test; Supplementary Table 1). Moreover, levels of ultrasensitive c-reactive protein (usCRP) in the GR group decreased after one-year treatment (*P* < 0.05 by the Wilcoxon signed-rank test), which indicates that inflammation in the GR group is possibly treated by supplementation with the probiotics. To further evaluate effects of supplementation with *L. reuteri* ATCC PTA 6475 on the gut microbiota and metabolomic profiles of the subjects, we collected fecal samples and serum samples from these 20 subjects at baseline and 12 months, respectively, as illustrated in Fig. 1a.

**Table 1:** Comparions of characteristics in the GR and PR groups at baseline and 12 months.

Characteristics	Baseline		12 months		*P*^1^	*P*^2^
	GR (*n* = 10)	PR (*n* = 10)	GR (*n* = 10)	PR (*n* = 10)		
Age (year)	76.6 ± 1.1	76.3 ± 1.0	77.6 ± 1.0	77.4 ± 1.0	0.57	0.56
Height (cm)	162.6 ± 5.1	163.1 ± 4.3	163.0 ± 5.5	163.2 ± 4.3	0.82	0.94
Weight (kg)	72.4 ± 8.2	63.9 ± 7.7	73.3 ± 8.5	64.0 ± 8.0	**0.03**	**0.02**
Body mass index (kg/m^2^)	27.5 ± 3.6	24.0 ± 2.8	27.7 ± 3.7	24.1 ± 3.1	**0.03**	**0.03**
Bone mineral density (g/cm^2^)						
Lumbar spine	0.99 ± 0.13	0.98 ± 0.1	1.01 ± 0.14^*^	0.98 ± 0.09	0.84	0.63
Total hip	0.82 ± 0.1	0.81 ± 0.05	0.82 ± 0.09	0.8 ± 0.05	0.76	0.60
Femoral neck	0.66 ± 0.07	0.68 ± 0.07	0.66 ± 0.07	0.66 ± 0.08^#^	0.67	0.92
HR-pQCT derived bone variables						
Total tibia vBMD (mg/cm^3^)	247 ± 39.2	231 ± 44.9	248 ± 39.8	226 ± 44.2^#^	0.42	0.26
Trabecular BV/TV (%)	13.1 ± 2.29	11.5 ± 1.98	13.1 ± 2.34	11.4 ± 2.11	0.12	0.10
Cortical vBMD (mg/cm^3^)	787 ± 46.5	769 ± 74.5	786 ± 44.6	758 ± 70.6^#^	0.52	0.30
Cortical thickness (mm)	0.84 ± 0.17	0.82 ± 0.23	0.84 ± 0.17	0.79 ± 0.23^#^	0.84	0.53
Serum markers						
N-terminal telopeptide (nM)	14.8 ± 3.97	13.1 ± 2.97	14.1 ± 2.35	14.8 ± 3.64	0.30	0.61
BAP (U/L)	16.4 ± 3.55	15.6 ± 2.97	17.2 ± 3.91	16.2 ± 2.14	0.60	0.52
usCRP (mg/L)	2.14 (1.53–3.68)	0.98 (0.8–2.47)	1.57 (1.13–1.90)^*^	1.36 (0.67–3.19)	0.25	0.91
TNF-α (pg/mL)	1.28 (1.09–1.57)	1.23 (0.98–1.47)	1.24 (1.11–1.59)	1.15 (1.04–1.35)	0.63	0.47
Body composition (kg)						
Total fat mass	28.9 (27.1–32.0)	20.8 (19.3–24.5)	28.2 (24.3–31.7)	20.0 (18.5–23.7)	**0.04**	**0.04**
Total lean mass	44.2 ± 3.2	41.4 ± 3.6	45.7 ± 3.6^*^	42.3 ± 4.1	0.09	0.06

Note: Mean ± SD. Non-normally distributed variables are presented as median with interquartile range. For comparison between the GR and PR groups at each time point, Student’s *t*-test or Wilcoxon rank sum test were used as appropriate. For comparison between baseline and 12 months in each group, paired *t*-test or Wilcoxon signed-rank test were used.

‘*’ and ‘#’ denote significant difference (*P* < 0.05) between baseline and 12 months in the GR and PR groups, respectively.

*P* ^1^ and *P* ^2^ values are derived from comparisons between the GR and PR groups at baseline and 12 months, respectively.

The significant differences (*P* < 0.05) are highlighted in bold.

*Total vBMD* Total tibia volumetric bone mineral density, *Trabecular BV/TV* Trabecular bone volume fraction, *Cortical vBMD* Cortical volumetric bone mineral density, *BAP* Bone-specific alkaline phosphatase, *usCRP* ultrasensitive c-reactive protein, *TNF-α* Tumor necrosis factor alpha.

### The overall composition of gut microbiota after one-year supplementation with *L. reuteri* ATCC PTA 6475

**Fig. 2:**
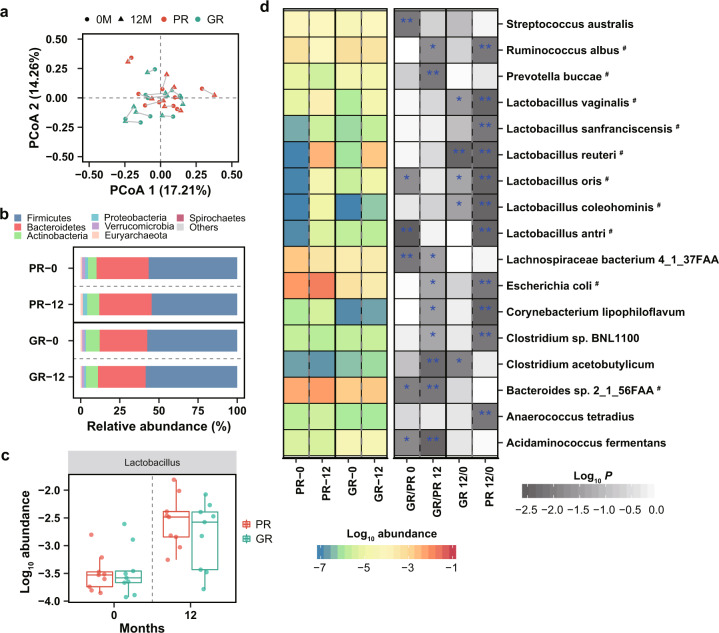
Compositional differences of the gut microbiota after supplementation with *L. reuteri* ATCC PTA 6475. **a** Principal coordinate analysis (PCoA) of microbiota community at species level based on Bray–Curtis distance. The overall microbial composition between the GR (*n* = 9) and PR (*n* = 9) groups or between the two time points shows no significant difference, estimated by PERMANOVA analysis. The blue and red colors indicate the GR and PR groups, respectively; The dot and triangle indicate the baseline (0 M) and 12 months (12 M), respectively. **b** Relative profiles of top seven abundant phyla. **c** Increased abundance of the genus *Lactobacillusin* in the GR (*n* = 9) and PR (*n* = 9) groups at 12 months (*P-*value < 0.01 by the Wilcoxon signed-rank test). The upper quartile, median and lower quartile are shown in the box plot. The blue and red colors indicate the GR and PR groups, respectively. **d** The left heatmap shows log-transformed mean abundances of differential species in the two groups at the two time points. The blue and red colors indicate the low and high abundances, respectively; The grey color in the right heatmap indicates *P*-value of comparative analysis using the Wilcoxon test**;** ‘*’ denotes *P* < 0.05; ‘**’ denotes *P* < 0.01; ‘#’ indicates the differential species were also identified by DESeq2 (adjusted *P*-value < 0.05 and |log_2_ fold change | >1.

To identify alteration of gut microbiota after *L. reuteri* ATCC PTA 6475 treatment and potential microbial signatures that are important for a good response to the probiotic, the fecal metagenomes of the selected 20 subjects were characterized using shotgun sequencing. The taxonomic profiles were first calculated using the MEDUSA pipeline^13^. Principal coordinate analysis (PCoA) and α diversity analysis results showed no significant differences in the overall microbial composition between the GR and PR groups at either baseline or 12 months (Fig. 2a and Supplementary Fig. 1). Also, there were no distinct shifts in the gut microbiota in the two groups after *L. reuteri* ATCC PTA 6475 treatment (Fig. 2a). Moreover, Fig. 2b showed the overview of microbial species abundances at phylum levels, mainly including Firmicutes, Bacteroidetes and Actinobacteria, which exhibited no significant differences between two groups or time points using the Wilcoxon test (Supplementary Table 2). Furthermore, we performed differential analyses of the taxonomic profiles at levels of class, order, family, genus and species between the GR and BR groups or between the two time points. Genus *Lactobacillus* and family Lactobacillaceae had elevated abundances after 12 months in the two groups (*P* < 0.01 by the Wilcoxon signed-rank test; Fig. 2c and Supplementary Fig. 2), but showed no difference between the GR and PR groups at each time point. Therefore, at high taxonomic levels, there were no significant differences in the microbial composition between the two groups or time points in this study, except for genus *Lactobacillus* and family Lactobacillaceae.

### Detrimental changes of microbial species in poor responders after one-year supplementation with *L. reuteri* ATCC PTA 6475

After one-year supplementation, species *L. reuteri* abundances were increased in the GR and PR groups (*P* < 0.01 by the Wilcoxon signed-rank test and adjusted *P* < 0.05 & |log_2_ fold change | > 1 by DESeq2; Fig. 2d and Supplementary Table 2 and 3), but did not show differences between the two groups. In total, we identified 16 species differential between the two groups or between the two time points in total (*P* < 0.01 by the Wilcoxon test; Fig. 2d). Four species, including *Prevotella buccae* (adjusted *P* < 0.05 by DESeq2), *Clostridium acetobutylicum*, *Bacteroides sp. 2_1_56FAA* (adjusted *P* < 0.05 by DESeq2), *Acidaminococcus fermentans*, had differential abundances between the two groups at 12 months, while three species, including *Streptococcus australis*, *Lactobacillus antri* (adjusted *P* < 0.05 by DESeq2), *Lachnospiraceae bacterium 4_1_37FAA*, showed differential between the two groups at baseline (*P* < 0.01 by the Wilcoxon rank-sum test; Fig. 2d). *Lactobacillus antri* was less abundant in the PR group than the GR group, while *Lachnospiraceae bacterium 4_1_37FAA* was more abundant in the PR groups at baseline (*P* < 0.01 by the Wilcoxon rank-sum test). Moreover, *Escherichia coli* was enriched in the PR group compared to the GR group at 12 months (adjusted *P* = 0.03 & log_2_ fold change = −3.8 by DESeq2, Supplementary Table 3).

In addition, 11 differential species, including *Escherichia coli*, were identified between the two time points in the PR group while only one differential species (*L. reuteri*) in the GR group (*P* < 0.01 by the Wilcoxon signed-rank test; Fig. 2d), which suggests that microbial shifts mainly happen in the PR group. *E. coli* was increased solely in the PR group (adjusted *P* = 0.04 & log_2_ fold change = 3.7 by DESeq2, Supplementary Table 3) and meanwhile exhibited different levels between the GR and PR groups at 12 months.

Also, we calculated relative abundances of species using MetaPhlAn2 pipeline^14^, and performed comparative analyses between the GR and PR groups or the two time points using the Wilcoxon test (Supplementary Table 4). Only four species *L. reuteri*, *Coprococcus catus*, *Gordonibacter pamelaeae* and *Erysipelatoclostridium ramosum* showed differential abundances between the GR and PR groups or between the two time points (*P* < 0.01 by the Wilcoxon test; Supplementary Fig. 3). *E. coli* was enriched, while *Akkermansia muciniphila*, *Ruminococcus bicirculans*, *Eubacterium_sp_CAG_38* and *Butyricimonas virosa* were depleted in the PR groups at 12 months, compared to the GR group (*P* < 0.05 by the Wilcoxon rank-sum test; Supplementary Fig. 3).

### Functional alterations of the gut microbiota after supplementation with *L. reuteri* ATCC PTA 6475

**Fig. 3:**
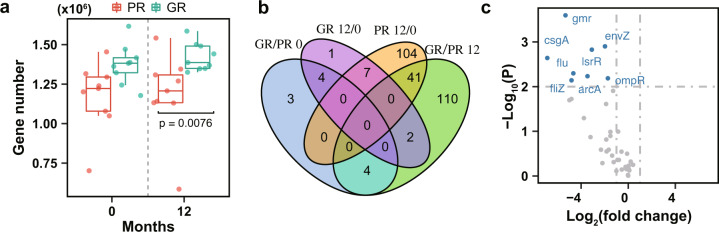
Potential function alterations of the gut microbiota after supplementation with *L. reuteri*. **a** Gene number of the gut microbiota in the PR (*n* = 9) and GR (*n* = 9) groups at the two time points. 20 million reads from each sample were sampled in order to rarefy the reads to the same depth of sequencing. The blue and red colors indicate the GR and PR groups, respectively. The upper quartile, median and lower quartile are shown in the box plot. **b** The Venn diagram shows differential KOs between the GR (*n* = 9) and PR (*n* = 9) groups or between baseline and 12 months (Adjusted *P*-value < 0.1 by DESeq2). ‘GR/PR 0’ and ‘GR/PR 12’ denote the comparative analysis between the GR and PR groups at baseline and 12 months, respectively. ‘GR 12/0’ and ‘PR 12/0’ denote the comparative analysis between the two time points in the GR and PR groups, respectively. **c** The volcano plot displays the differential genes involved in biofilm formation (*Escherichia coli*) between the GR (*n* = 9) and PR (*n* = 9) groups at 12 months. The blue color indicates the differential genes; The horizontal and vertical dashed lines indicate *P-*value < 0.01 and |log_2_ fold change | >1, respectively.

The functional capacities of the gut microbiome in the GR and PR groups were investigated at baseline and 12 months, respectively. We first calculated the gene and KO (KEGG Orthology) profiles using the MEDUSA pipeline^13^. In comparison to the PR group, gene richness had an increased trend in the GR group at baseline but not statistically significant (Fig. 3a). Interestingly, gene richness was significantly higher in the GR group (1.40 ± 0.11 million) than the PR group (1.18 ± 0.26 million) at 12 months (*P* < 0.01 by the Wilcoxon rank-sum test; Fig. 3a). By comparative analysis, there were 11 and 157 KOs identified to be significantly differential between the two groups at baseline and 12 months, respectively (Adjusted *P* < 0.1 by DESeq2; Fig. 3b; Supplementary Table 5). This suggests that functional capacities of the endogenous baseline microbiota show no significant difference between the GR and PR groups. In addition, 152 differential KOs were identified between the two time points in the PR group while only 14 differential KOs in the GR group (Adjusted *P* < 0.1 by DESeq2; Fig. 3b), indicating that microbial function altered in the PR group over time much more than the GR group.

Furthermore, by the gene set analysis (GSA), there were 16 differentially enriched KEGG pathways identified by comparing the GR and PR groups at 12 months or by comparing the two time points in the PR group (*P* < 0.05 by GSA; Supplementary Table 6). Interestingly, the microbial metabolism related to biofilm formation (*E. coli*) was enriched in the PR group at 12 months in comparison to baseline (*P* < 0.05 by GSA; Supplementary Table 6). The abundances of microbial genes involved in biofilm formation, including *gmr*, *arcA*, *lsrR*, *rcsC* and *rcsD*, were increased in the PR group (*P* < 0.01 by DESeq2 and |log_2_ fold change > 1 | ; Supplementary Fig. 4). Accordantly, we observed difference in the metabolism related to biofilm formation (*E. coli*) between the GR and PR groups at 12 months (*P* < 0.05 by GSA; Supplementary Table 6). The genes including *arcA*, *gmr*, *csgA* and *envZ* involved in biofilm formation, were more abundant in the PR group than the GR group after one-year treatment (*P* < 0.01 by DESeq2 and |log_2_ fold change > 1 | ; Fig. 3c). The capacity of microbial phenylalanine metabolism was also increased in the PR group at 12 months compared to the baseline (*P* < 0.05 by GSA; Supplementary Table 6).

Meanwhile, we calculated the relative profiles of the MetaCyc pathways using the HUMAnN2 tool^15^, and performed comparative analyses between the GR and PR groups or the two time points (Supplementary Table 7). In agreement with results of the GSA, the enrichment of superpathway of L − phenylalanine biosynthesis was observed in the PR group at 12 months (*P* < 0.01 by the Wilcoxon signed-rank test; Supplementary Fig. 5). In addition, the capacity of polyisoprenoid biosynthesis (*E. coli*) was elevated in the PR group at 12 months compared to baseline (*P* < 0.05 by the Wilcoxon signed-rank test; Supplementary Fig. 6).

### Metabolomics changes in response to the supplementation with *L. reuteri* ATCC PTA 6475

**Fig. 4:**
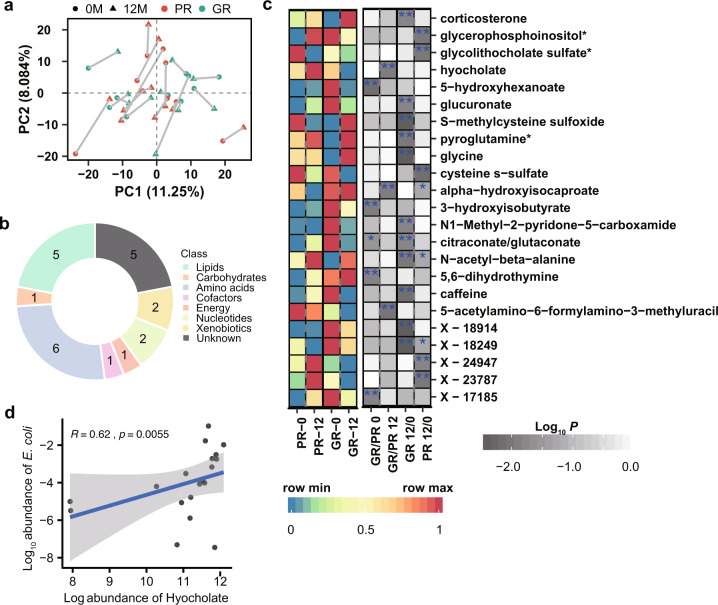
Changes of metabolic profiles of the older women after treatment with *L. reuteri*. **a** The score plot of principal components analysis (PCA) based on metabolomic profiles in the GR (*n* = 10) and PR (*n* = 10) groups at baseline and 12 months. The blue and red colors indicate the GR and PR groups, respectively; The dot and triangle indicate the baseline (0 M) and 12 months (12 M), respectively. **b** Biological processes in which the differential metabolites are involved (*P* < 0.01 by the Wilcoxon test). **c** The left heatmap shows scaled mean abundances of the differential metabolites in the PR (*n* = 10) and GR (*n* = 10) groups at two time points. The blue and red colors indicate the low and high abundances, respectively; The grey color in the right heatmap indicates *P*-value of comparative analysis; ‘*’ denotes *P* < 0.05; ‘**’ denotes *P* < 0.01. **d** Association between metabolite hyocholate and the species *E. coli*. The blue line and grey shade indicate the regression line and 95% confidence interval; *R* denotes Spearman’s correlation coefficient.

Early studies have revealed that the gut microbiota could impact bone metabolism by producing SCFAs, which can stimulate bone formation^11^. To investigate the effects of *L. reuteri* ATCC PTA 6475 on global metabolism of the 20 subjects in the GR and PR groups, we analyzed 40 serum metabolomics profiles collected at baseline and 12 months from these women in our previous study^16^. In total, the metabolomics dataset comprised of 1,232 metabolites that were measured by liquid chromatography-tandem mass spectroscopy (LC-MS). Principal component analysis (PCA) showed that metabolomic profiles did not exhibit a clear distinction between the GR and PR groups at baseline or 12 months (Fig. 4a).

Furthermore, we performed differential analysis of metabolite abundances between the two groups or between the two time points (Supplementary Table 8), and a total of 23 metabolites were identified to be differentially abundant (*P* < 0.01 by the Wilcoxon test), which are mainly involved in biological processes lipids and amino acids (Fig. 4b). Amino acids have been suggested to be associated with bone metabolism^17^. In accordance, six amino acids derived metabolites were identified to be differential in this study (*P* < 0.01 by the Wilcoxon test; Fig. 4b). Out of them, three metabolites, including hyocholate, alpha−hydroxyisocaproate, 5−acetylamino−6−formylamino−3−methyluracil, had differential abundances between the two groups at 12 months, while four metabolites, including 5−hydroxyhexanoate, 3−hydroxyisobutyrate, 5,6−dihydrothymine, showed differential between the two groups at baseline (*P* < 0.01 by the Wilcoxon rank-sum test; Fig. 4c). Hyocholate, involved in secondary bile acid metabolism, had an increased abundance in the PR group at 12 months, compared to the GR group (*P* < 0.01 by the Wilcoxon rank-sum test).

Moreover, 11 and 5 metabolites abundances differed between the two time points in the GR and PR groups, respectively (*P* < 0.01 by the Wilcoxon signed-rank test; Fig. 4c). The abundance of cysteine s−sulfate decreased in the PR group after one-year probiotic supplementation (*P* < 0.01 by the Wilcoxon signed-rank test). Cysteine s-sulfate is a structural analog of glutamate and could act as an agonist of N-methyl-D-aspartate receptor (NMDA-R)^18^, which was reported to be involved in bone resorption^19^. Interestingly, butyrylcarnitine (C4) showed an elevated trend in the GR group after probiotic supplementation (*P* < 0.05 by the Wilcoxon signed-rank test; Supplementary Fig. 7 and Supplementary Table 8), which is consistent with our early report that butyrylcarnitine (C4) was increased in older women supplemented with *L. reuteri* ATCC PTA 6475 compared to placebo^16^. Butyrylcarnitine (C4) could act as the pool and transporter of butyrate^20^, which has previously been shown to inhibit bone resorption and stimulate bone formation in mice^11,21,22^.

To explore the potential links between the differential metabolites and species, we performed correlation analyses at baseline and 12 months, respectively (Supplementary Table 9). At 12 months, we observed associations between 5,6-dihydrothymine and *Prevotella buccae* (*R* = 0.61, *P* = 0.0056, Supplementary Table 9), between citraconate/glutaconate and *Ruminococcus albus* (*R* = −0.72, *P* = 0.00049) and between S-methylcysteine and *Lactobacillus.coleohominis* (*R* = −0.58, *P* = 0.0087). Particularly, metabolite hyocholate showed a positive correlation with species *E. coli* at 12 months (*R* = 0.62, *P* = 0.0055; Fig. 4d). Moreover, at baseline we found associations between glycolithocholate sulfate* and *Bacteroides sp.2_1_56FAA* (*R* = 0.71, *P* = 0.00068, Supplementary Table 9), between pyroglutamine* and *Prevotella buccae* (*R* = 0.58, *P* = 0.01) and between 3-hydroxyisobutyrate and *Acidaminococcus fermentans* (*R* = 0.6, *P* = 0.007).

## Discussion

Daily supplementation of *L. reuteri* ATCC PTA 6475 was reported to substantially reduce bone loss in older women in our recent study^9^. Here we first selected 20 elderly women with the best or poorest responses to the treatment with *L. reuteri* ATCC PTA 6475 from the previous PP population. Except for differences in weight and BMI, subjects in the GR and PR groups had no significant differences in the clinical characteristics at baseline, but presented with significant differences in reducing bone loss after one-year probiotic treatment. This indicates that we were successful in sampling a population with similar baseline characteristics, which to some extent eliminates confounding factors for studying the effects of supplementation with *L. reuteri* ATCC PTA 6475 on bone metabolism and gut microbiota. Intriguingly, in the GR group supplementation with *L. reuteri* ATCC PTA 6475 decreased levels of the serum inflammation marker ultrasensitive CRP that has been reported to be associated with reduced BMD^23,24^. In line with this, a previous study suggested that supplementation with *L. reuteri* ATCC PTA 6475 decreased intestinal inflammation and increased bone density in healthy male mice^25^.

In this study, significant differences in the microbial composition at high taxonomic levels were not observed between the groups or time points, except for genus *Lactobacillus*. However, at species levels, there are statistical differences. At baseline, several species (*Streptococcus australis*, *Lactobacillus antri*, *Lachnospiraceae bacterium 4_1_37FAA, C. catus*) and KOs were identified to be differential between the GR and PR groups. The difference in BMI between the GR and PR groups might associate with the discrepancies in the endogenous baseline microbiota, which could be important for the different probiotic effects in good responders versus poor responders. One previous report showed that the baseline microbiota differed for participants who responded with increased insulin sensitivity index after *L. reuteri* supplementation, and was characterized by higher microbial diversity^26^.

Multiple studies have suggested that the gut microbiota could be affected by probiotic supplementation^10,11^, which would regulate bone mass via effects on the immune system^3,5,27^. Various probiotic strains including *Lactobacillus rhamnosus* and *Bifidobacterium longum* have been shown to influence bone formation through the production of SCFAs^6,11,28^. Here we observed that one-year supplementation with *L. reuteri* ATCC PTA 6475 resulted in an altered gut microbiome, including a significant increase of gene richness in the GR group. Several SCFAs-producing bacterial species, including *C. acetobutylicum*, *A. fermentans*, *A. muciniphila, C. catus* and *R. bicirculans* show more abundant in the GR group than the PR group at 12 months. *C. acetobutylicum*, *R. bicirculans* and *C. catus* have been reported to be able to produce SCFAs by fermenting dietary carbohydrates^29–32^. Moreover, *A. fermentans* was suggested to be able to ferment glutamate to butyrate^33^. Previous studies have showed that *A. muciniphila* has the ability to degrade mucins in the intestine mainly into SCFAs, which have promising probiotic activities^34–36^. Thus, our results show that probiotic treatment enhances the abundances of these SCFAs-producing bacterial species in the GR group, which might contribute to the reduction of bone loss.

In addition, detrimental changes in the microbial species and function in the poor responders were alleviated in the good responders. *E. coli* is more abundant in the PR group at 12 months in comparison to the GR group. There is growing evidence that colonization with *E. coli* could induce expression of pro-inflammatory cytokines and subsequently cause intestinal inflammation and barrier disruption^37–39^. Moreover, previous studies have implicated that the intestinal dysbiosis potentially contribute to osteoporosis in the elderly via the immune system^40,41^. Consistently, by functional analysis we found that the capacity of *E. coli* biofilm formation was increased in the PR group at 12 months. It suggests that *E. coli* could be more adherent to the gut mucosa in the PR group, which may be more favorable to elicit inflammation^42^. Therefore, decreased abundance of *E. coli* and increased abundance of the SCFAs-producing bacterial species in the GR group after treatment with *L. reuteri* ATCC PTA 6475 indicates that this results in establishment of a more beneficial intestinal ecosystem and barrier function, which could reduce inflammation and improve bone traits. This is in agreement with an early study proposing that probiotic *L. reuteri* ATCC PTA 6475 could prevent bone loss through reducing intestinal dysbiosis and barrier disruption^43^.

As previously described, an appropriate sample size enables a microbiome research to discern the differences between groups^44^. It should be noted that the number of elderly women recruited in this study was small with 20 subjects (ten good responders and ten poor responders), which could have influenced our results. Therefore, future investigations with a larger sample size would further increase the statistical power and improve the generalizability of our results. Moreover, our ability to identify differential metabolites and species could have been affected by multiple comparisons. Another potential limitation is the absence of subjects from the placebo group. Current study only explored the potential differences between the good responders and poor responders. However, the effects of *L. reuteri* supplementation on the gut microbiota in elder women is still unclear due to the absence of comparison between probiotics group and the placebo group. Therefore, the introduction of subjects of placebo in the comparison will advance our understanding of the effects of probiotics on the gut microbiota related to bone loss.

In conclusion, we observed a significantly increased gene richness, enrichment of SCFAs producing species as well as depletion of *E. coli* and its biofilm formation in the GR group, by investigating alterations of the gut microbiome during 1 year *L. reuteri* ATCC PTA 6475 supplementation. This indicates that *L. reuteri* ATCC PTA 6475 supplementation might promote bone formation by modulating the gut microbiota composition and function, which could be crucial for the development of novel osteoporosis treatments. However, further studies are needed to validate the mechanisms of how the altered gut microbiota is linked to bone metabolism.

## Methods

### Study population

From the per protocol (PP) population in the placebo-controlled cohort where 68 elderly women had been randomized to supplementation with the probiotic strain *L. reuteri* ATCC PTA 6475 or placebo^9^, 20 elderly women with bone loss who supplemented with probiotic *L. reuteri* ATCC PTA 6475 were selected for the following secondary analysis. Out of these women, ten women had the highest changes (good responders, GR) and the lowest changes (poor responders, PR) in tibia total volumetric BMD after one-year supplementation with *L. reuteri* ATCC PTA 6475, respectively. These 20 women with different responses to the treatment with *L. reuteri* ATCC PTA 6475 completed the study and had not used medication in violation of the study protocol. The study was approved by the Regional Ethics Committee in Gothenburg, D-nr 075-15 (26 February 2015) and addendum T229-16 (10 March 2016). The study was registered at Clinicaltrials.gov (number NCT02422082) prior to study start. All participants provided written informed consent to take part in the study.

### Serum sample preparation and measurements of serum markers

Serum samples were isolated from whole blood taken from the cubital vein of the selected 20 elderly women at baseline and 12 months as described in our recent study^16^. These samples were frozen immediately after collection and stored in −80 °C until used in the further measurement assays. A 500 ul aliquot of serum was used for analysis of serum markers. N-telopeptide of type I collagen (NTx; Osteomark®; Alere Scarborough, Inc., Scarborough, ME, USA; intra- and inter-assay variability 4.6 and 6.9%, respectively), bone-specific alkaline phosphatase (BAP; MicrovueTM; Quidel Corporation, Athens, OH, USA; intra- and inter-assay variability 5.0 and 5.9%, respectively), ultrasensitive C-reactive protein (usCRP; BioMerica, Inc., Irvine, CA, USA; intra and inter-assay variability 4.4 and 3.3%, respectively) and tumour necrosis factor-α (TNF-α; Quantikine®; R&D Systems, Inc., Minneapolis, MN, USA; intra- and inter-assay variability 2.0 and 6.7%, respectively) were measured in duplicate by TECO medical AG (Sissach, Switzerland).

### Measurements of bone traits and body composition

The lower leg (tibia) of the non-dominant side (based on dominant arm) was measured to determine bone microstructure and geometry, using High Resolution peripheral Computed Tomography (HR-pQCT; Xtreme CT, Scanco Medical AG, Brüttisellen, Switzerland). All measurement procedures were performed according to instructions and protocols from the manufacturer as previously described^45,46^. A 3D construction of the bone was assembled using 110 slices, obtained from each measurement. The nominal isotropic resolution was 82 μm. After image processing^47^, the following variables were obtained: total volumetric BMD (total vBMD, mg/cm^3^), trabecular bone volume fraction (BV/TV, %), cortical thickness (Ct.Th, mm), and cortical volumetric bone mineral density (Ct.vBMD, mg/cm^3^). Image quality was assessed based on qualitative observations of movement artifacts, including blurring or streaks in reconstructed 2D slices, and was graded from one to five, with a perfect quality was defined as one and a suboptimal quality was five. Only images with a quality level between one and three were used for analysis. Areal BMD (aBMD, g/cm^2^) was measured at the lumbar spine (L1-L4), total hip and femoral neck and body composition of the total body with a Hologic Discovery A dual x-ray absorptiometer (DXA; Hologic, Waltham, MA, USA).

### Fecal sample preparation and metagenomic profiling

We collected 19 fecal samples at baseline and 12 months respectively, from the 20 elderly women in the GR and PR groups. In each group, nine individuals have time points-paired fecal samples. Fecal samples were frozen immediately after collection and stored in −80 °C until analysis. Total fecal genomic DNA was extracted from 100 mg feces using a modification of the IHMS DNA extraction protocol Q^48^. Briefly, fecal samples were extracted in Lysing Matrix E tubes (MP Biomedicals) containing ASL buffer (QIAGEN), and lysis of cells was obtained, after homogenization by vortexing for 2 min, by two cycles of heating at 90 °C for 10 min each followed by three bursts of bead beating at 5.5 m/s for 60 s in a FastPrep®-24 Instrument (MP Biomedicals). After each bead-beating burst, samples were placed on ice for 5 min. The supernatants containing fecal DNA were collected after the two cycles by centrifugation at 4 °C. Supernatants from the two centrifugations steps were pooled and a 600 µL aliquot from each sample was purified using the QIAamp DNA Mini kit (QIAGEN) in the QIAcube (QIAGEN) instrument using the procedure for human DNA analysis. Samples were eluted in 200 µL of AE buffer (10 mmol/L Tris·Cl; 0.5 mmol/L EDTA; pH 9.0). Libraries for shotgun metagenomic sequencing were prepared by a PCR-free method; library preparation and sequencing were performed at Novogene (Nanjing, China) on a NovaSeq instrument (Illumina Inc.) with 150 bp paired-end reads. The average read count per sample are 24.7 ± 1.4 million.

Tool Kneaddata (https://github.com/biobakery/kneaddata) was first used to perform quality control on the metagenomic sequencing data with parameters –trimmomatic Trimmomatic-0.36 --trimmomatic-options “SLIDINGWINDOW:4:20 MINLEN:50” -t 9 --bowtie2-options “--very-sensitive --dovetail”. The sequencing runs had high quality with almost 96% of the reads passing the quality cut-off. Out of the high-quality reads, on average 0.03% were aligned to the human genome. Then, tools MetaPhlAn2 that relies on unique clade-specific marker genes^14^, and HUMAnN2^15^ were used to profile the composition and function of the gut microbiota from the quality filtered reads. MetaPhlAn2 and HUMAnN2 were run with default parameters and following databases: mpa_v293_CHOCOPhlAn used for microbial taxon profiling; UniRef90 gene clusters for quantifying genes/proteins; MetaCyc in conjunction with the UniRef90 gene clusters for quantifying pathway abundances. Relative abundances of species at different taxonomy levels and MetaCyc pathways were used for downstream analyses.

In addition, pipeline MEDUSA that was developed to process raw shotgun metagenomics sequence data^13^, was performed to profile the microbial composition and genes/KEGG Orthology (KOs). Out of the quality filtered no-human reads, average 79% were mapped to the MEDUSA’s software gene catalogue that contains >11 million genes, and average 45% were mapped to genome catalogue that contains 1747 species genomes, using tool Bowtie2^49^. Read counts at different taxonomy levels were normalized by scaling with cumulative sum (i.e. relative abundance). To determine the richness of the microbiota using the gene catalogue, two aligned reads were required to indicate a gene present in a sample as used in the previous study^13^, and all samples were rarefied to the same number of reads, 20 million, in order to eliminate the effect of different sequencing depth.

### Metabolomic profiling

Approximately 500 ul aliquot of serum was used for the metabolome assay. Metabolomics were performed using liquid chromatography-tandem mass spectroscopy (LC-MS) at Metabolon, Inc (Morisville, NC, USA) with their standard and platform method^50^. The metabolomics dataset comprised a total of 1,232 metabolites with 958 compounds of known identity (named metabolites) and 274 compounds of unknown structural identity (unnamed metabolites).Peak intensities of all metabolites in original scale (provided by Metabolon, Inc) were analyzed. First, we used an imputation method drawing random values from the normal distribution to simulate signals from low abundant metabolites that were assumed to give rise to missing values^51^. The width of the normal distribution was set as 0.5 times of the standard deviation (SD) of all measured values and the center was down-shifted by 2.5 times of the SD. Second, metabolites with <25% missing values across all samples were selected and analyzed further, which enabled identification of important metabolites with high confidence. Finally, peak intensities of the filtered metabolites within sample *i* (*i* = *1*, *2*, *3*…*n*, *n* is the total number of samples) were normalized to the total intensity. This normalization method considered the differences of sample volume loaded onto LC-MS and assumes that all samples have equal total intensity^52^. The normalization process included the following steps: (a) The total intensity of sample *i* was summed up; (b) Correction factor of sample *i* was calculated by dividing the total intensity with the lowest total intensity of all samples; (c) Peak areas of all metabolites within sample *i* were divided by its individual correction factor. After data normalization, we obtained abundances of 993 metabolites with <25% missing values for downstream analyses. The normalized metabolite abundance was log transformed in this study.

### Statistical analysis

To visualize and evaluate differences in gut microbiota composition between the two groups at two time points, principal coordinates analysis (PCoA) and PERMANOVA (R function adonis) were performed based on species-level Bray–Curtis distances. The α diversity of the gut microbiota was calculated based on species-levels of each sample using Shannon, Simpson and Invsimpson indices via R package vegan^53^. To identify the differential KOs and species, multivariate negative binomial generalized linear models were performed by R package DESeq2^54^, using raw read counts from the MEDUSA pipeline. Only genes or species with the sum of counts across all samples ≥10 and existed in at least five samples were considered in the analysis. Raw read counts of KOs and species were normalized using the median of ratios method by DESeq2. To further explore differences in KEGG functions, gene set analysis (GSA) was performed using Piano with the reporter algorithm for KEGG pathways based on fold changes and *P*-values of KOs^55^. The differentially enriched KEGG pathways were identified with a distinct directional *P*-value < 0.05.

To identify differential signatures including metabolites, microbial taxon at different levels and MetaCyc pathways, we used the two-tailed Wilcoxon rank-sum test to compare the GR and PR groups at each time point, and used Wilcoxon signed-rank test to compare the time points-paired samples in each group. The Spearman’s rank correlation analysis was performed to assess the associations between metabolites and species. Principal components analysis (PCA) was performed to visualize differences in serum metabolomic profiles between the GR and PR groups or between the two time points. Adjusted *P-*values were obtained by using the false discovery rate method (Benjamini–Hochberg) to control for multiple comparisons error.

## Supplementary information

supplementary materials

Supplementary Table 2–9

## Data Availability

The dataset supporting the conclusions of this manuscript is available in the European Nucleotide Archive (ENA) repository under the accession number PRJEB52923.

## Code availability

Code and scripts used in the analyses are available upon request.
