# Supplementary material for: One-year supplementation with Lactobacillus reuteri ATCC PTA 6475 counteracts a degradation of gut microbiota in older women with low bone mineral density
Source: NPJ Biofilms Microbiomes. 2022 Oct 19;8:84. doi: 10.1038/s41522-022-00348-2 (PMC9581899; doi:10.1038/s41522-022-00348-2)
Supplement: Supplementary file 1 — supplementary materials [file 41522_2022_348_MOESM1_ESM.pdf]

Supplementary Materials

Supplementary Tables

Supplementary Table 1. Relative changes in clinical variables of the GR and PR groups.

**Characteristics**	**GR (n = 10)**	**PR (n = 10)**	***P*** [a]
Height	0.27 ± 0.32	0.07 ± 0.29	0.17
Weight	1.25 ± 2.47	0.1 ± 2.44	0.31
Body mass index	0.71 ± 2.3	-0.04 ± 2.7	0.51
Bone mineral density			
Lumbar spine	1.94 ± 2.57	0.5 ± 2.28	0.2
Total hip	0.44 ± 1.49	-0.47 ± 1.49	0.19
Femoral neck	-0.87 ± 2.46	-2.47 ± 3.14	0.22
HR-pQCT derived bone variables			
Total tibia volumetric bone mineral density	0.39 ± 0.77	-2.22 ± 0.58	**< 0.001**
Trabecular bone volume fraction	0.26 ± 0.89	-0.99 ± 2.11	0.11
Cortical volumetric bone mineral density	-0.17 ± 0.95	-1.43 ± 1.0	**0.01**
Cortical thickness	0.72 ± 1.39	-4.07 ± 2.3	**< 0.001**
Serum markers			
N-terminal telopeptide	-0.9 ± 18.9	16.5 ± 30.4	0.14
Bone-specific alkaline phosphatase	5.94 ± 19.5	5.78 ± 15.6	0.98
C-reactive protein	-13.9 ± 44.1	12.5 ± 53.1	0.24
Tumor necrosis factor alpha	-0.31 ± 9.95	10.1 ± 26.5	0.27
Body composition			
Total fat mass	-3.28 ± 4.92	-3.4 ± 5.3	0.96
Total lean mass	3.55 ± 3.13	2.24 ± 4.54	0.46

Note: Mean ± SD.

[a] *P* value were derived from Student's t-test. The characteristics significantly different ($P < 0.05$) between GR and PR groups were highlighted in bold.

Supplementary Table 2. Comparisons of microbial taxa at phylum (a) and species (b) levels between the GR and PR groups or between the two time points. The taxonomic profiles were calculated using the MEDUSA pipeline. The two-tailed Wilcoxon rank-sum test was used to compare the GR and PR groups at each timepoint, and the Wilcoxon signed-rank test was used to compare the timepoints-paired samples in each group (n=9).

Supplementary Table 3. Differential analysis of microbial species between the GR and PR groups or between the two time points, performed by DESeq2.The taxonomic profiles were calculated using the MEDUSA pipeline.

Supplementary Table 4. Comparisons of microbial t species between the GR and PR groups or between the two time points. The taxonomic profiles were calculated using the MetaPhlAn2 pipeline. The two-tailed Wilcoxon rank-sum test was used to compare the GR and PR groups at each timepoint, and the Wilcoxon signed-rank test was used to compare the timepoints-paired samples in each group (n=9).

Supplementary Table 5. Differential analysis of KEGG Orthology (KO) profiles between the GR and PR groups or between the two time points, performed by DESeq2. The KO profiles were calculated using the MEDUSA pipeline.

Supplementary Table 6. The gene set analysis (GSA) of KEGG pathways by comparing the GR and PR groups at 12 months or by comparing the two timepoints in the PR group using fold changes and *P* values of KOs.

Supplementary Table 7. Differential analysis of Metacyc pathways between the GR and PR groups or between the two time points. The Metacyc pathway profiles were calculated using the HUMAnN2 pipeline. The two-tailed Wilcoxon rank-sum test was used to compare the GR and PR groups at each timepoint, and the Wilcoxon signed-rank test was used to compare the timepoints-paired samples in each group (n=9).

Supplementary Table 8. Comparative analysis of metabolites profiles between the GR and PR groups or between the two time points. The two-tailed Wilcoxon rank-sum test was used to compare the GR and PR groups at each timepoint, and the Wilcoxon signed-rank test was used to compare the timepoints-paired samples in each group (n=10).

Supplementary Table 9. Associations between the differential metabolites and species at baseline (a) and 12 months (b), respectively. The Spearman's rank correlation analysis was performed.

Supplementary Figures

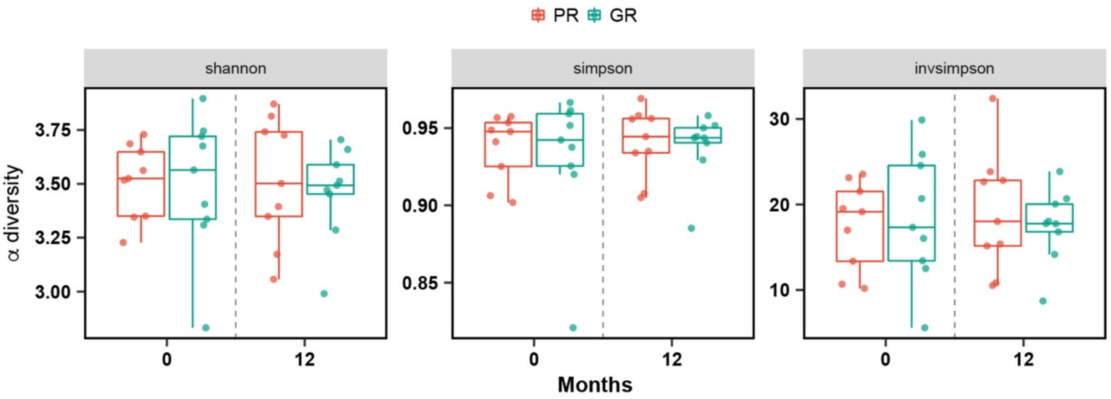

Supplementary Figure 1. Comparisons of α- diversity between the GR and PR groups or between the two time points. The two-tailed Wilcoxon rank-sum test was used to compare the GR and PR groups at each timepoint, and the Wilcoxon signed-rank test was used to compare the timepoints-paired samples in each group (n=9).

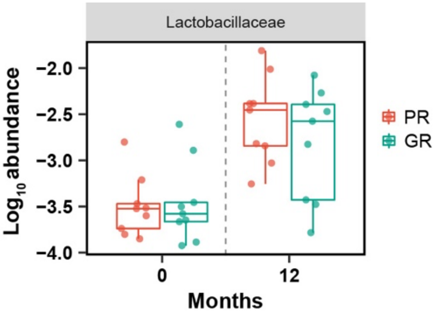

Supplementary Figure 2. Comparisons of family Lactobacillaceae abundance between the GR and PR groups or between the two time points. The two-tailed Wilcoxon rank-sum test was used to compare the GR and PR groups at each timepoint, and the Wilcoxon signed-rank test was used to compare the timepoints-paired samples in each group (n=9).

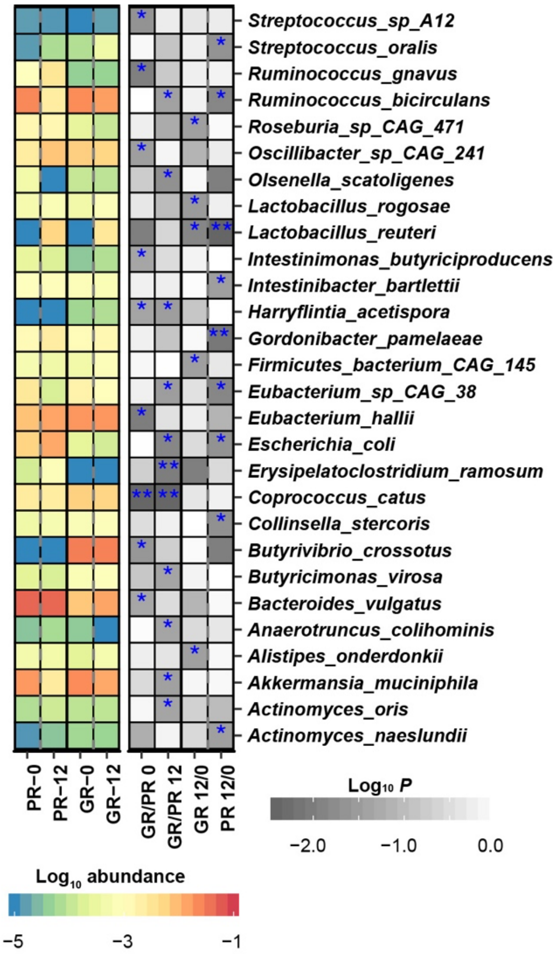

Supplementary Figure 3. The left heatmap shows log-transformed mean abundances of differential species in the two groups at the two time points. The two-tailed Wilcoxon rank-sum test was used to compare the GR and PR groups at each timepoint, and the Wilcoxon signed-rank test was used to compare the timepoints-paired samples in each group (n=9). The species profiles were calculated using the MetaPhlAn2 pipeline.

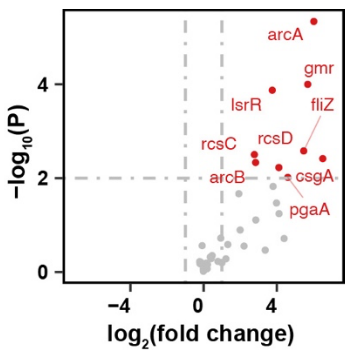

Supplementary Figure 4. The volcano plot displays differential KOs involved in biofilm formation (*Escherichia coli*) between baseline and 12 months in the PR groups. The horizontal and vertical dashed lines indicate *P* value < 0.01 and |log2 fold change| >1.

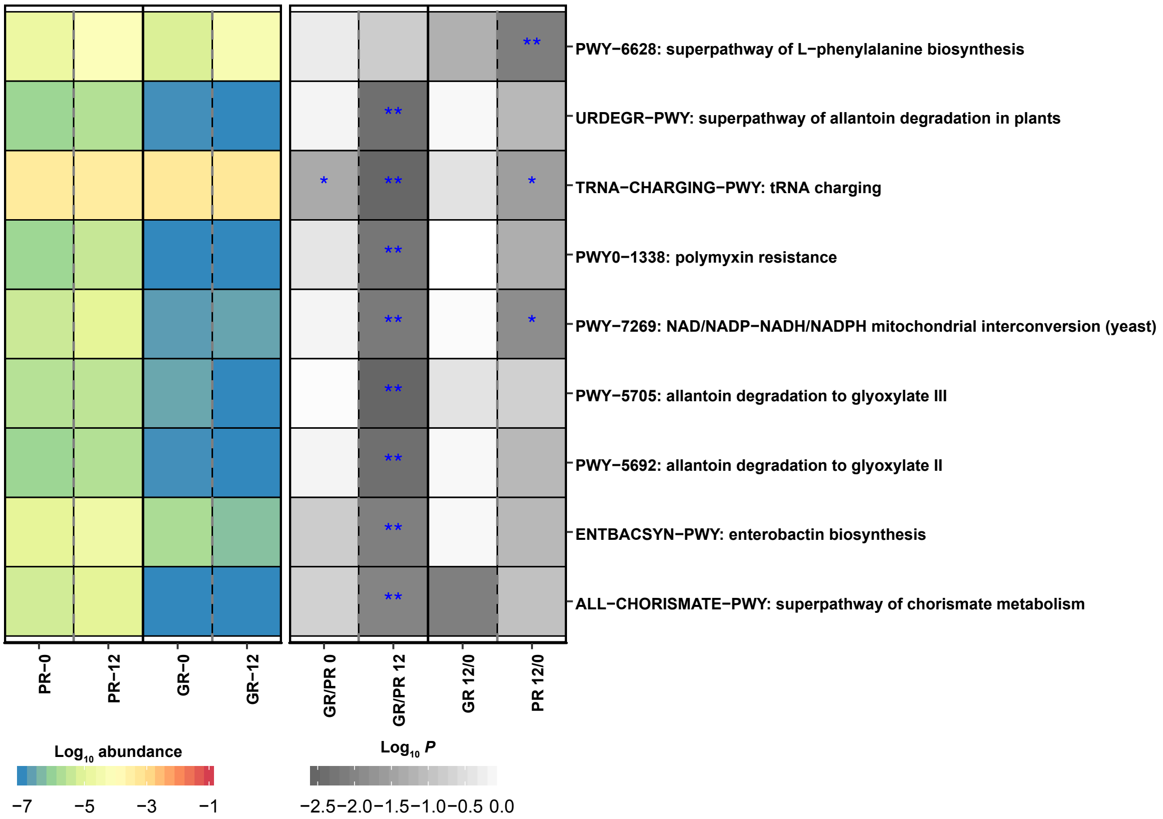

Supplementary Figure 5. The left heatmap shows log-transformed mean abundances of differential MetaCyc pathways in the two groups at the two time points. The grey color in the right heatmap indicates *P* value of comparative analysis; '*' denotes $P < 0.05$; '**' denotes $P < 0.01$. The two-tailed Wilcoxon rank-sum test was used to compare the GR and PR groups at each timepoint, and the Wilcoxon signed-rank test was used to compare the timepoints-paired samples in each group (n=9).

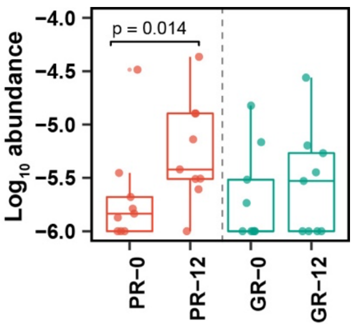

Supplementary Figure 6. Comparisons of polyisoprenoid biosynthesis (*E. coli*) between the GR and PR groups or between the two time points. The two-tailed Wilcoxon rank-sum test was used to compare the GR and PR groups at each timepoint, and the Wilcoxon signed-rank test was used to compare the timepoints-paired samples in each group (n=9).

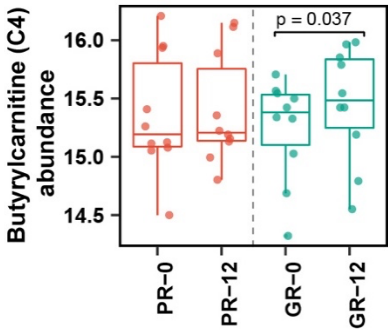

Supplementary Figure 7. Comparisons of butyrylcarnitine (C4) between the GR and PR groups or between the two time points. The two-tailed Wilcoxon rank-sum test was used to compare the GR and PR groups at each timepoint, and the Wilcoxon signed-rank test was used to compare the timepoints-paired samples in each group (n=10).
